# Differential effects of disease modifying drugs on peripheral blood B cell subsets: A cross sectional study in multiple sclerosis patients treated with interferon-β, glatiramer acetate, dimethyl fumarate, fingolimod or natalizumab

**DOI:** 10.1371/journal.pone.0235449

**Published:** 2020-07-27

**Authors:** C. L. Kemmerer, V. Pernpeintner, C. Ruschil, A. Abdelhak, M. Scholl, U. Ziemann, M. Krumbholz, B. Hemmer, M. C. Kowarik

**Affiliations:** 1 Department of Neurology & Stroke, and Hertie-Institute for Clinical Brain Research, Eberhard-Karls University of Tübingen, Tübingen, Germany; 2 Department of Neurology, Klinikum rechts der Isar, Technische Universität München, Munich, Germany; 3 Munich Cluster for Systems Neurology (SyNergy), Munich, Germany; Institut Cochin, FRANCE

## Abstract

**Background:**

Several disease modifying drugs (DMDs) have been approved for the treatment of multiple sclerosis (MS), however, little is known about their differential impact on peripheral blood (PB) B cell subsets.

**Methods:**

We performed a cross sectional study on PB B cells in MS patients treated with interferon-β (n = 25), glatiramer acetate (n = 19), dimethyl fumarate (n = 15), fingolimod (n = 16) or natalizumab (n = 22), untreated MS patients (n = 20), and in patients with non-inflammatory neurological diseases (n = 12). Besides analyzing routine laboratory data, flow cytometry was performed to analyze naïve B cells (CD19+CD20+CD27-IgD+), non-class switched (CD19+CD20+CD27+IgD+) and class-switched memory B cells (CD19+CD20+CD27+IgD-), double negative B cells (CD19+CD20lowCD27-IgD-) and plasmablasts (CD19+CD20lowCD27+CD38++).

**Results:**

Treatment associated changes were found for the overall B cell pool as well as for all B cell subsets. Natalizumab increased absolute numbers and percentage of all B cells mainly by expanding the memory B cell pool. Fingolimod decreased absolute numbers of all B cell subsets and the percentage of total B cells. Fingolimod, dimethyl fumarate and interferon-β treatments were associated with an increase in the fraction of naïve B cells while class switched and non-class switched memory B cells showed decreased percentages.

**Conclusion:**

Our results highlight differential effects of DMDs on the PB B cell compartment. Across the examined treatments, a decreased percentage of memory B cells was found in dimethyl fumarate, interferon-β and fingolimod treated patients which might contribute to the drugs’ mode of action in MS. Further studies are necessary to decipher the exact role of B cell subsets during MS pathogenesis.

## Introduction

Multiple lines of evidence have indicated that B cells play an important role in the pathogenesis of multiple sclerosis (MS). Beside the persistence of intrathecal oligoclonal bands and detection of B cells within MS lesions, B cell depleting therapies have been shown to be highly effective [1]. Moreover, various MS treatments exert differential effects on B cell subsets but the exact roles of B cells during the different drugs’ mode of action remain inconclusive.

Analyses of peripheral blood (PB) B cell subsets during the course of MS show partially inconsistent results, depending on the definition of B cell subsets, disease course, clinical activity and age of patients [2, 3]. With the exception of one study [4], several studies have shown an increased percentage of naïve B cells and decreased percentage of memory B cells in the peripheral blood, especially during relapse [3, 5, 6]. As a possible explanation it has been proposed, that memory B cells are directed to the site of inflammation in active disease [5]. Indeed, increased values of mainly memory B cells and plasmablasts are found in the cerebrospinal fluid (CSF) which persist during MS disease course [5, 7, 8]. However, B cell trafficking across the blood-brain-barrier and B cell maturation within the CNS show complex patterns [9, 10] and the precise involvement of the different B cell subsets in MS pathology still remains unclear.

In this study we performed a cross sectional analysis of PB B cell subsets in MS patients receiving interferon-β (IFN-β), glatiramer acetate (GLAT), dimethyl fumarate (DMF), fingolimod (FTY) or natalizumab (NAT). We found differential effects on multiple B cell subsets with a marked decrease of memory B cells in several treatments.

## Materials and methods

### Standard protocol approvals and patients

Patients were recruited at the Departments of Neurology at the Universitätsklinikum Tübingen and the Klinikum rechts der Isar of the Technische Universität München. All participants consented to the usage of their biological samples for research purposes. The study was approved by the ethics committee of the medical faculty of the Universität Tübingen and Technische Universität München. MS patients visiting the MS center in Munich between 2015 and 2017 and patients visiting the MS center in Tübingen between 2018 and 2019 were recruited for our study. Study inclusion criteria for the MS patients were the following: 1) diagnosis of clinically definite MS according to the 2017 [11] and 2010 [12] McDonald criteria 2) the ability to give informed consent; and 3) stable disease at clinical visit. Exclusion criteria included 1) CNS disease in addition to MS; 2) primary progressive form of MS; 3) relapse within 60 days prior to clinical visit; 4) severe bacterial or viral infection within the last 30 days. 20 patients with MS were untreated, 22 MS patients received NAT treatment, 15 MS patients received DMF, 16 MS patients received FTY, 19 MS patients were treated with GLAT and 25 MS patients were treated with IFN-β. In addition, 12 patients with non-inflammatory neurological diseases including headache (2), visual deficit of unknown origin (2), paresthesia of unknown origin (2), trigeminal neuralgia (1), stroke (1), hypoesthesia (1), normal pressure hydrocephalus (1), cervical spine disorder (1) and neuropathic pain (1) served as controls. Initiation of new treatments after previous immunomodulatory therapy followed the German guidelines for the treatment of multiple sclerosis and implied the normalization of previous treatment specific effects (e.g. on differential blood counts). Further patient characteristics are summarized in Table 1 and shown in detail in the S1 Table.

**Table 1:** Patient characteristics.

Patient group	Number of patients	Age (median, in years (range))	Sex (male: female)	disease duration (median, in months)	EDSS (Median (range))	Therapy duration (median, in months)	Previous Immuno-modulatory therapy	Concommitant medications (MS associated)
NIND	12	51.5 (21–76)	2:10	-	-	-	none	
UT	20	35.5 (18–66)	5:15	38.5	1.75 (0–2.5)	-	IFN (N = 4), GLAT (= 1), DMF (N = 1)	Escitalopram, pregabalin, vitamin D, simvastatin, citalopram
NAT	22	37 (18–58)	1:21	96.5	2 (0–6)	16	IFN (N = 8), GLAT (N = 8), DMF (N = 3), FTY (N = 10), DACL (N = 1), AZA (N = 1), TER (N = 1)	Citalopram, escitalopram, mirtazapine, amitriptyline, paroxetine, olanazpine, baclofen, pregabalin, vitamin D, 4-aminopyridine
DMF	15	5.5 (26–51)	3:12	42	1.5 (0–3)	27	IFN (N = 7), GLAT (N = 3), Ter (N = 1)	Duloxetine, vitamin D,
FTY	16	38 (23–58)	11:5	79	2 (0–3.5)	20	GLAT (N = 6), DMF (N = 3), IFN (N = 9), NAT (N = 6)	vitamin D, gabapentin, vitamin B12, pregabalin
GLAT	19	36.5 (26–61)	6:13	51	1 (0–4.5)	25	IFN (N = 7), DMF (N = 2)	Doxepine, citalopram, vitamin D, gabapentin, trimipramine, paroxetine, levetriracetam, vitamin B12
IFN	25	38 (23–64)	11:14	66	1 (0–3.5)	36	GLAT (N = 2) NAT (N = 1)	vitamin D, escitaliopram

Abbreviations: NIND = non-inflammatory neurological diseases, UT = untreated MS patients, NAT = natalizumab, DMF = dimethyl fumarate, FTY = fingolimod, GLAT = glatiramer acetate, IFN = interferon-β, DACL = Daclizumab, TER = Teriflunomid, AZA = Azathioprine, EDSS = Expanded Disability Status Scale.

Absolute values of the B cell populations were calculated from the percentages and the absolute lymphocyte counts if available from routine diagnostics in the respective hospitals using the following equation:

$$absolute\;count\;target\;population=\frac{absolute\;count\;lymphocytes*\;percentage\;target\;population\;}{100}.$$

Patients whos sample acquisition of differential blood count and peripheral blood count differed were not considered for absolute lymphocyte count analysis (N = 17).

### Specimen handling

Samples were processed as follows: 1,6ml of whole blood was collected in Monovette EDTA tubes (Sarstedt, Germany) and analyzed 24 hours post collection latest. 100l blood of each patient was collected in a 96-well plate (Greiner Bio-one, Germany) and diluted 1:1 with phosphate buffered saline (Dulbecco’s PBS, Sigma Aldrich, United Kingdom). After centrifugation for 4 minutes at 4°C and 400g samples were lysed twice with 180μl ACK buffer (aqueous solution of 150mM NH4Cl (Sigma Aldrich, Germany), 10mM KHCO3 (Merck KGaA, Germany) and 0.1mM EDTA (Merck KGaA, Germany) adjusted to pH = 7,3) for ten minutes. Cells were washed with FACS buffer (phosphate buffered saline (Dulbecco’s PBS, Sigma Aldrich, Germany), 2% fetal bovine serum (Gibco, life technologies, Germany), 0.01% NaN3 (Serva GmbH, Germany and Karl Roth GmbH, Germany) and 2mM EDTA (Merck KGaA, Germany) and incubated with the monoclonal antibody mix for 30 minutes at 4°C in the dark. The samples were washed again and resuspended in FACS buffer for analysis. Flow cytometry was performed on the CyAn ADP (Dako, Agilent Technologies). Samples from the Technische Universität München were processed as described above with minor differences in the composition of buffers and a shorter incubation time of monoclonal antibodies [13].

### Cell staining and definition of B cell subsets

The antibody mix consisted of CD38(HB7) fluorescein isothiocyanate (FITC, Becton, Dickinson and Company, BD Biosciences, USA), polyclonal rabbit anti-human IgD/RPE Phycoerythrin (PE, Dako, Agilent Technologies, Denmark), CD19 Phycoerythrin-Texas Red-X (ECD, Beckman Coulter Company, France), CD20 (L27) Allophycocyanin-Cyanin7 (APC Cytm7, Becton, Dickinson and Company, BD Biosciences, USA), CD3 (SK7) Phycoerythrin-Cyanin 7 (PE Cytm7, Becton, Dickinson and Company, BD Biosciences, USA), CD45 V450 mouse anti-human clone H130 (Becton, Dickinson and Company, BD Biosciences, USA) and CD27 (L128) Allophycocyanin (APC, Becton, Dickinson and Company, BD Biosciences, USA) for all samples. Antibodies were all diluted 1:15 with FACS buffer except from CD38 FITC, which was diluted 1:5.

B cell subtypes were defined similar as by Kowarik et al [14] with the following surface markers: Naïve B cells (CD19+CD20+CD27-IgD+), non-class-switched (CD19+CD20+CD27+IgD+) and class-switched memory B cells (CD19+CD20+CD27+IgD-), double negative B cells (CD19+CD20lowCD27-IgD-) and plasmablasts (CD19+CD20low CD27++ CD38++). Gates were adjusted for sample variability and the experimenter was blinded for the treatment conditions of the samples during gating.

### Data analysis

Flow cytometry data was analyzed using Summit Software (Version 4.3 for Windows, Dako Cytomation) and Microsoft Excel (Version 2016 for Windows, Microsoft Corporation). For statistical analysis and graphical images SPSS (IBM Corp. Released 2017. IBM SPSS Statistics for Windows, Version 25.0. Armonk, NY: IBM Corp.) and for graphical images GraphPad Prism (Version 5 for Windows, GraphPad Software, San Diego, California USA) and Microsoft PowerPoint (Version 2016 for Windows, Microsoft Corporation) were used.

We first analysed our data set for potential effects of confounders that could influence the statistical analysis between the different patient groups. The distribution of gender in our data set was tested by Chi2 test (nominal scale data, χ2(6) = 22.450) and revealed an uneven distribution of gender in our patient cohort. Except for fingolimod which is reported to cause slightly lower lymphocyte counts in women [15], no gender specific effects on lymphocytes are reported for other treatments to the best of our knowledge. Since require-ments for parametric data were fulfilled for the analysis of age (interval scale data, Kolmo-gorov-Smirnoff-test for normality and Levene’s test for equality of error variances not signif-icant), we conducted an ANOVA test which showed no significant effect of age as a possible confounder. To test whether differences in the samples from the Technische Universität München and the Universität Tübingen could account for differences in the B cell subsets we conducted a nonparametric Mann-Whitney-U tests where group sample sizes where larger than five patients. We could not detect differences between the specific B cell subsets (p > 0.05).

We next analysed the data for statistical differences between the various patient groups. After significant deviations from normality were detected in the samples for the immune cell populations (Kolmogorov-Smirnoff test p < 0.05), the non-parametric Kruskal-Wallis test was used to find differences in immune cell populations between all treatment groups. Multiple comparisons were adjusted by Bonferroni correction and the significance level was set to p ≤ 0.05.

## Results

### Absolute lymphocyte, neutrophile, monocyte, eosinophile and basophile counts under MS treatments

**Fig 1:**
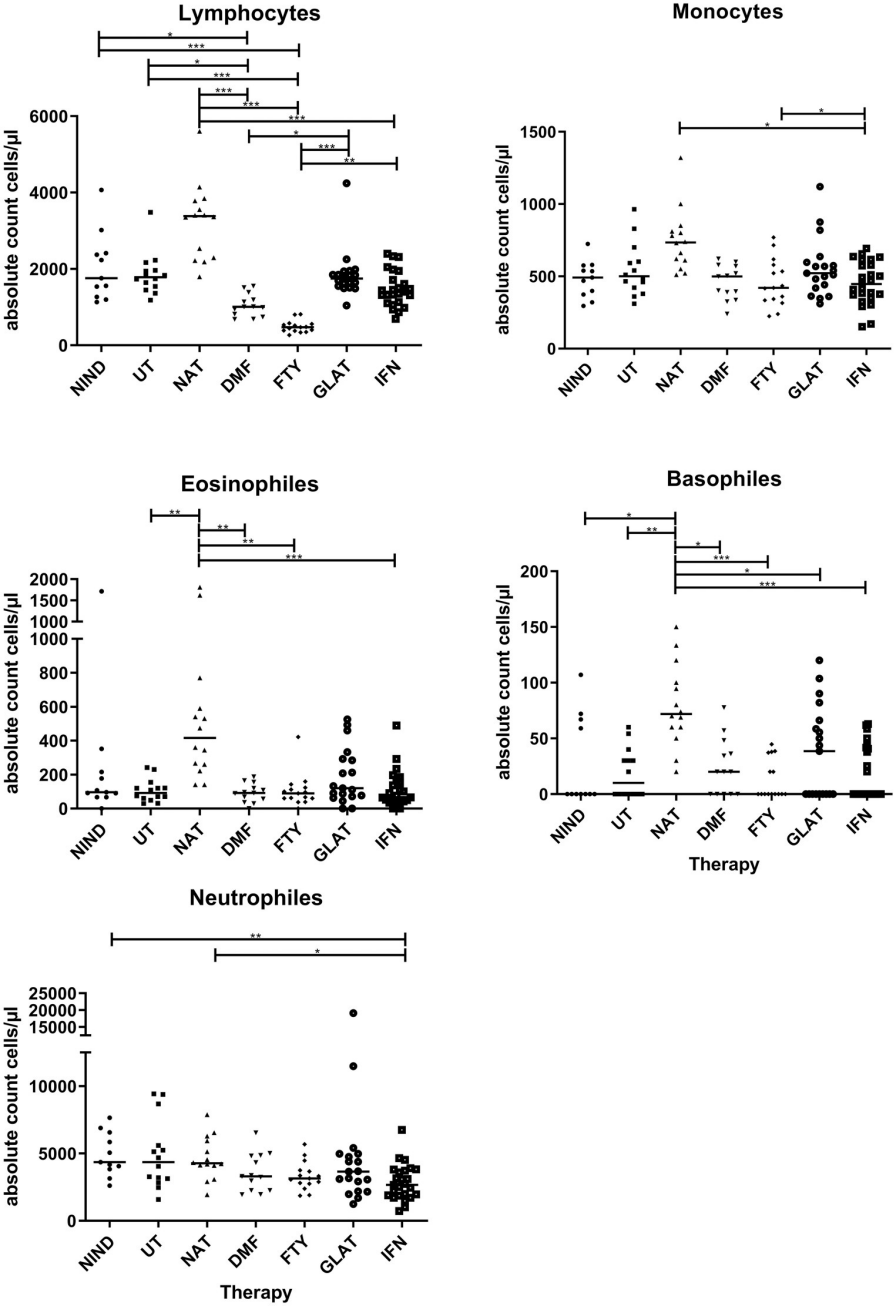
Absolute lymphocyte, neutrophil, monocyte, eosinophil and basophil counts. Displayed are the absolute counts for lymphocytes, neutrophils, monocytes, eosinophils and basophils for different treatments (NIND = non-inflammatory neurological disease, UT = untreated MS patients, NAT = natalizumab treated MS patients, DMF = dimethyl fumarate treated MS patients, FTY = fingolimod treated MS patients, GLAT = glatiramer acetate treated MS patients and IFN-ß = interferon-β treated MS patients). Lines in the graphs indicate median values and asterisks describe significant differences (Kruskal-Wallis test with Bonferroni correction) as follows: (*) p ≤ 0.05, (**) p < 0.01, (***) p < 0.001.

We first analyzed differential blood counts assessed during the routine diagnostic work up of our patients. We did not observe significant differences within the immune cell subsets between patients with NIND and untreated MS patients (Fig 1). Comparing MS patients receiving DMDs to untreated MS patients, absolute lymphocyte counts were reduced in DMF (p = 0.042) and FTY (p < 0.001) treated patients. NAT treated patients showed elevated eosinophil (p = 0.003) and basophil (p = 0.002) counts compared to untreated MS patients while IFN treated patients showed a trend towards reduced neutrophil counts (p = 0.054). A detailed overview on additional significant differences of immune cell counts between the various treatments is shown in Fig 1.

### Absolute counts of B cell subsets under MS treatments

**Fig 2:**
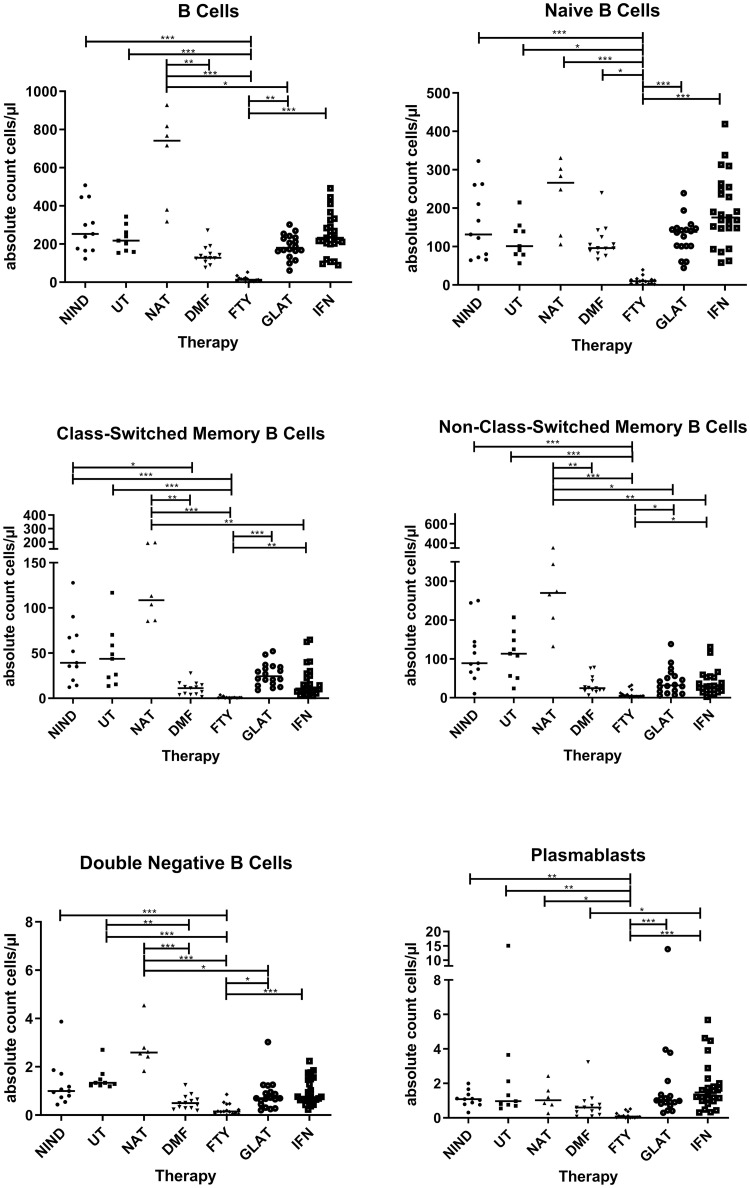
Absolute B cell counts. Displayed are the absolute counts of B cell populations for different treatments (NIND = non-inflammatory neurological disease, UT = untreated MS patients, NAT = natalizumab treated MS patients, DMF = dimethyl fumarate treated MS patients, FTY = fingolimod treated MS patients, GLAT = glatiramer acetate treated MS patients and IFN-ß = interferon-β treated MS patients). Lines in the graphs indicate median values and asterisks describe significant differences (Kruskal-Wallis test with Bonferroni correction) as follows: (*) p ≤ 0.05, (**) p < 0.01, (***) p < 0.001.

We next calculated absolute numbers of different B cell subsets by referring the percentage distribution of B cells (assessed by flow cytometric analysis) to absolute lymphocyte counts. Again, no significant differences were observed between patients with NIND and untreated MS patients (Fig 2). Absolute B cell counts were reduced for all B cell subsets in FTY treated patients when compared to untreated MS patients (p<0.001 for total B cells, p = 0.048 for naïve B cells, p<0.001 for class-switched, non-class-switched memory B cells and double negative B cells, and p = 0.001 for plasmablasts). Additionally, DMF treatment was associated with a reduced number of double negative B cells (p = 0.007) when compared to untreated MS patients. NAT treated MS patients displayed elevated total B cell and memory B cell counts but these effects did not reach statistical significance. Further details on additional significant differences between treatments can be found in Fig 2.

### Percentage distribution of B cell subsets under MS treatments

**Fig 3:**
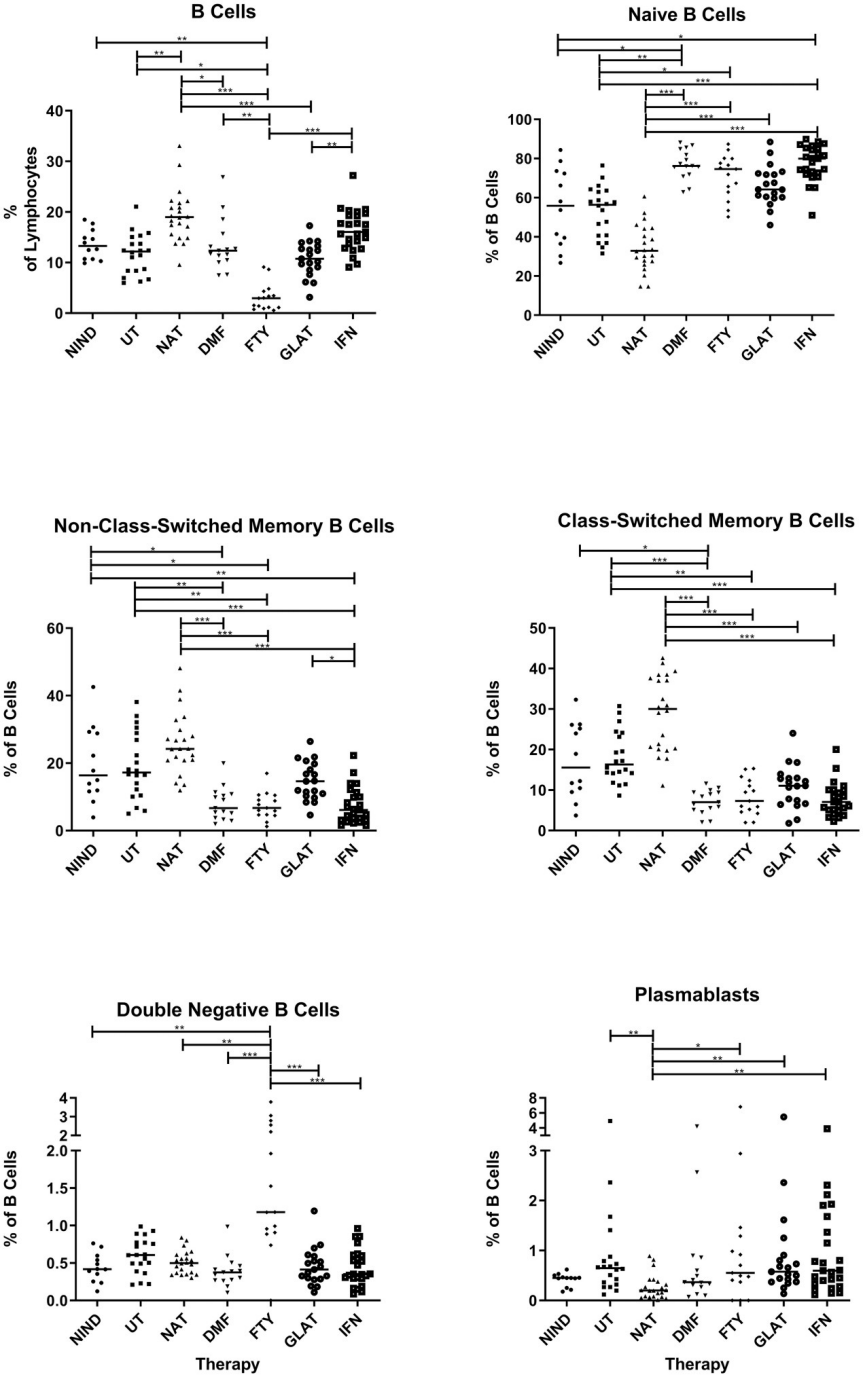
Percentage distribution B cell populations under different treatments. Displayed are the percentages of B cells of all lymphocytes and the percentages of B cell subpopulations relative to all B cells for different treatments (NIND = non-inflammatory neurological disease, UT = untreated MS patients, NAT = natalizumab, DMF = dimethyl fumarate, FTY = fingolimod, GLAT = glatiramer acteate and IFN = interferon-β treated MS patients). Lines in the graphs indicate median values and asterisks describe significant differences (Kruskal-Wallis test with Bonferroni correction) as follows: (*) p ≤ 0.05, (**) p < 0.01, (***) p < 0.001.

**Fig 4:**
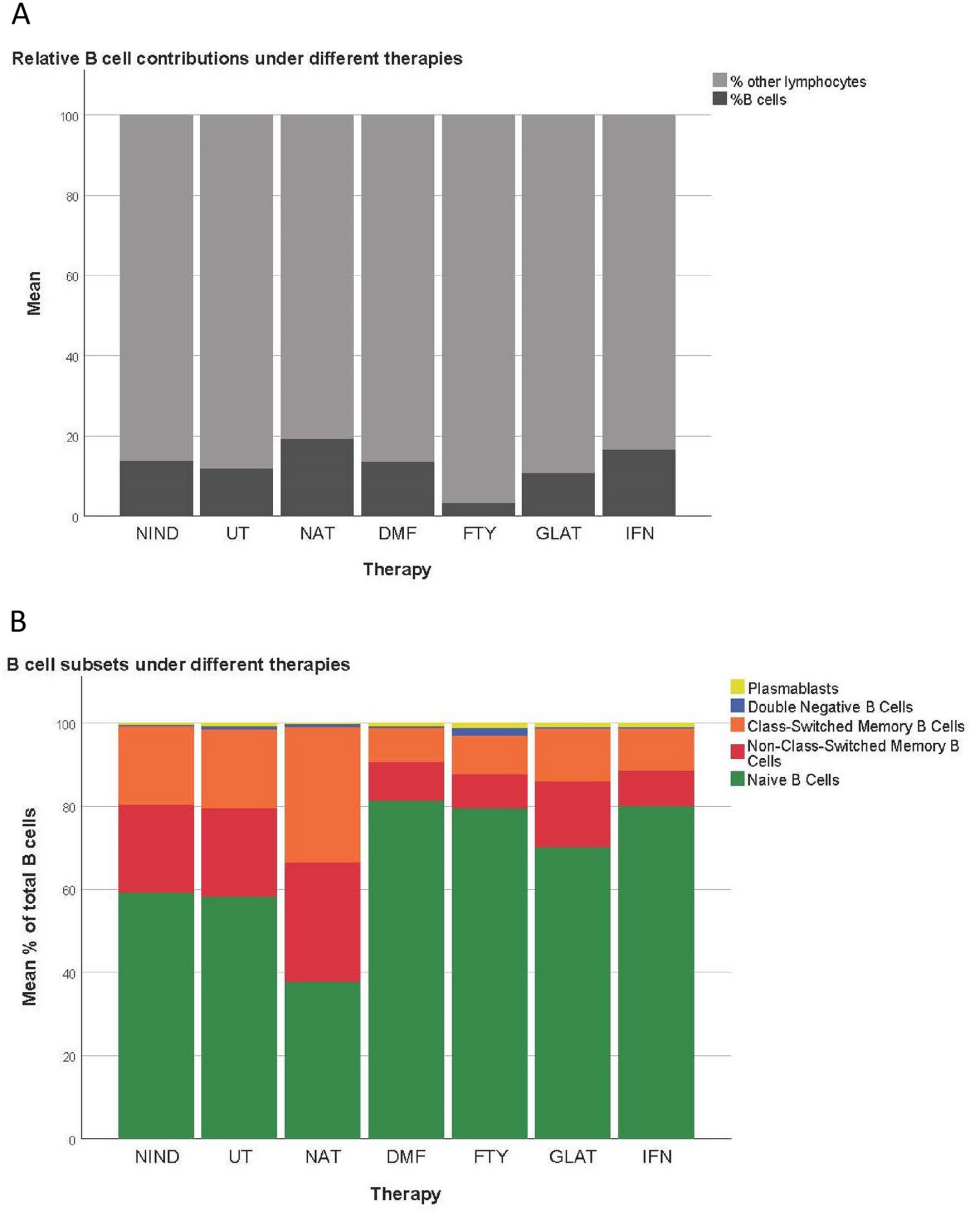
Relative B cell contributions under different therapies. A Displayed are the percentages of CD19+ B cells among the total circulating lymphocyte pool (gated events). B Displayed are the percentages of the circulating B cell subsets among the total CD19+ B cell pool (NIND = non-inflammatory neurological disease, UT = untreated MS patients, NAT = natalizumab, DMF = dimethyl fumarate, FTY = fingolimod, GLAT = glatiramer acteate and IFN = interferon-β treated MS patients).

Subsequently, the percentages of B cell subsets were assessed in comparison to untreated MS patients (Fig 3). Total B cell percentages were increased in NAT (p = 0.001) treated patients and decreased in FTY (p = 0.01) treated MS patients. Within the B cell pool, DMF, FTY and INF-β treated patients showed increased percentages of naïve B cells (p = 0.001, p = 0.041 and p < 0.001 respectively) and reduced percentages of non-class-switched (p < 0.001, p = 0.004 and p < 0.001 respectively) and class-switched memory B cells (p = 0.009, p = 0.002 and p < 0.001 respectively). Concerning plasmablasts, only NAT treated patients showed a significantly lower percentage compared to untreated MS patients (p = 0.005). NAT treated patients also showed a tendency towards reduced naïve B cells and elevated memory B cells, however, differences did not reach significance. Detailed significant differences between treatments groups are further shown in Fig 3; an overview on all B cell subsets is given in Fig 4.

## Discussion

The aim of this study was to characterize the composition of immune cells with focus on B cell subpopulations in the peripheral blood of MS patients treated with INF-β, GLAT, DMF, FTY or NAT. Treatment effects were investigated in a parallel approach during stable disease course in order to avoid inter-study discrepancies and immunological effects during relapse or additional immunomodulation with corticosteroids. We found differential effects on different B cell subsets, however, MS patients treated with DMF, FTY and INF-β showed a consistent percentage increase in naïve B cells whereas memory B cell percentages were significantly decreased (Fig 3). Although direct conclusions regarding functional aspects of B cells cannot be drawn from our analysis, our study provides deeper insights into effects on the B cell compound besides the basic understanding of the drugs’ mode of action.

Regarding injectible therapies, interferon-β (IFN-β) is believed to act via the modulation of cytokine production and the inhibition of leukocyte proliferation and antigen presentation [16] while glatiramer acetate (GLAT), among other effects, potentially competes with myelin basic protein (MBP) and other myelin antigens to bind and affect antigen presenting cells [17]. The precise mode of action of dimethyl fumarate (DMF) is currently unclear but a reduction of blood lymphocytes (including B cells) by induction of apoptosis and the modulation of cytokine expression have been proposed as potential mechanisms [18]. Fingolimod (FTY) is a functional antagonist of the sphingosine-1-phosphate receptor 1 expressed on lymphocytes and inhibits the egress of lymphocytes out of secondary lymphoid tissues [19] resulting in decreased circulating B cells numbers. Lastly, natalizumab (NAT) is an antibody against the cell adhesion molecule α4 integrin preventing trafficking of lymphocytes including B cells across the blood-brain barrier into the CNS [20, 21].

It has been well established that the percentage and absolute numbers of total B cells and memory B cells are elevated in NAT treated MS patients [22, 23]. In line with these results, we also observed an increase in relative B cell numbers and a trend towards increased absolute total B cell numbers and memory B cell counts and percentages. The effect of elevated total B cells most likely results from NAT’s mode of action, leading to an impaired trafficking of lymphocytes across the blood-brain barrier and a consecutive retention of lymphocytes including B cells in the peripheral blood [21]. The increased proportion of memory B cells can be explained by a release of marginal zone memory B cells through blocking of the physiological α4 integrin mediated adhesion to the marginal zone of the spleen [23, 24]. Regarding memory B cells, it could additionally be shown that the expression of CD49d (α4 integrin) was significantly higher in memory compared to naïve B cells [23]. This could further explain the less consistent results reported on the naïve B cell subset which should be less affected by the drug. In this study we found a trend towards reduced percentages of naïve B cells most likely due to an increased proportion of memory B cell percentages since absolute naïve B cell counts remained stable. Additionally, we could show that the percentage of plasmablasts was lower in NAT treated patients compared to untreated MS patients. Again, we could not find a change in the absolute number of plasmablasts in the B cell pool, so that this effect may also be attributed to the increased proportion of memory B cells in the peripheral blood. Moreover, NAT therapy was associated with an increased count of eosinophils and basophiles and a trend towards elevated monocytes. This effect has already been reported before [25, 26], however Miller and collegues found that values returned to baseline by month 9 during treatment. This is surprising, since most of the patients receiving NAT in our study have been treated for longer than 9 months. The reason for the increase in monocyte and eosinophil numbers is not finally unraveled, but also seems to resemble an α4 integrin dependent mechanism [27].

Concerning DMF, we found an increased percentage in naïve B cells along with a relative reduction of memory B cells which is in line with the literature [22, 28]. Absolute counts of double negative B cells were significantly decreased while memory B cells also showed a trend towards reduced numbers. This probably results from a differential apoptotic activity of DMF on lymphocyte subsets [29]. Li et al. showed, that DMF more strongly affected mature B cells, which could account for the relative decrease of memory B cells [18]. We could not replicate results from Diaz et al. who additionally found the percentage of double negative B cells to be decreased [29].

Treatment with FTY has been associated with a reduction of lymphocyte counts in the peripheral blood with an increased percentage of naïve B cells and a reduced percentage of memory B cells [22]. Our results support this finding but additionally showed an increased percentage of DN B cells. The reduction of memory B cells seems to happen preferentially for activated memory B cells [29] possibly contributing to the therapeutic efficacy of FTY. Furthermore, Nakamura et al. demonstrated that plasmablasts and monocytes express lower levels of S1P1, the receptor targeted by FTY to cause lymphocyte trapping in secondary lymphoid organs [30]. It is therefore surprising that plasmablast absolute counts were still significantly decreased along with all other B cell subsets in our analysis which points towards a similar effect of FTY on all B cell subsets.

It has been reported that GLAT reduces the relative frequency of B cells, plasmablasts and memory B cells in the peripheral blood of MS patients [31, 32]. We could only detect slight differences in relative B cell subset frequencies and GLAT seems to have a less profound effect on B cell subsets when compared to other DMDs. Potentially, the therapeutic efficacy of GLAT also relies on other immunological effects than a quantitative change in the B cell composition itself [17].

While absolute B cell numbers did not show significant effects after IFN-β treatment we detected an increased naïve B cell frequency and a reduced memory B cell frequency. Nevertheless, a visible trend towards a reduced memory B cell count supports evidence from Rizzo et al. stating that FAS-mediated apoptosis caused the decline in memory B cell numbers after IFN-β therapy [33]. These changes probably contribute to the effectiveness of IFN-β therapy.

We found differential changes in the frequency and number of B cell subsets for most DMDs, however, FTY, DMF and IFN-β treated patients were all associated with reduced memory B cell frequencies in the peripheral blood. Similarly, pulsed immune reconstitution therapies for MS such as cladribine and alemtuzumab have also been associated with significantly reduced memory B cell numbers over a prolonged time interval [34, 35]. Even though the decreased percentage of memory B cells is achieved by different mechanisms (retention, apoptosis), the relevance of targeting memory B cells for effective immunotherapy in MS has recently been a matter of debate [36]. The aforementioned treatments may have further modes of action, but the relative reduction of memory B cells may be associated with therapeutic efficacy.

Further evidence for the relevance of memory B cells in MS comes from clinical studies of the drug atacicept [36, 37]. Atacicept is believed to reduce B cell numbers in MS patients but specifically spares progenitor and memory B cells. Increased relapses in clinical trials were recorded and this worsening of the disease course potentially depended on an increased percentage of memory B cells. Nevertheless, alternative explanations cannot be ruled out. Another argument in favor of memory B cell related disease mechanisms comes from studies examining auto-proliferation of CD4+ T cells in MS [38, 39]. It has been shown that memory B cells of some multiple sclerosis patients induced proliferation of CD4 T cells targeting CNS self-antigens while memory B cells of healthy controls did not support this effect. Memory B cells therefore seem to have an important role in antibody-independent pathophysiological mechanisms.

In contrast to DMF, FTY and IFN-β, NAT showed completely different effects on peripheral blood B cells. Since its main mechanism is believed to prevent lymphocyte trafficking into the CNS, the distribution of B cell subtypes in the peripheral blood does not allow direct insights in the composition of intrathecal B cell subsets. Increased numbers of lymphocytes in the peripheral blood point towards a successful retention of these cells in the blood, however wash-out effects likewise increasing blood lymphocyte counts have to be disentangled from these effects. Indeed, two studies reported decreased CSF B cell counts in NAT treated patients [40, 41] supporting the hypothesis of an effective blocking of B cell trafficking across the blood-brain barrier.

Although memory B cells have been linked to MS disease pathology and populate the MS CNS together with plasmablasts and plasma cells [8] the exact functional properties during neuro-inflammatory cascades remain unclear. One possible pathophysiological aspect for memory B cells could be their recruitment from the peripheral blood into inflammatory CNS regions with further maturation into antibody secreting cells, antigen presentation to T cells and cytokine production. Indeed, peripheral blood class-switched memory B cells as well as plasmablasts and DN B cells were found to be clonally related to B cells within the CSF compartment, indicating, that B cell trafficking across the blood-brain barrier and further maturation within the CNS occurs in multiple sclerosis [9]. Thus, the reduction of circulating memory B cells or the retention of memory B cells within secondary lymphoid tissues or within the peripheral blood compartment itself could possibly interrupt the recruitment and migration of memory B cells to the site of inflammation. Furthermore, it could be shown that CNS B cells drain into cervical lymph nodes and recirculate into the CNS with a substantial maturation taking place in the periphery [10]. In this context, memory B cells that possibly encountered an antigen within the CNS could re-circulate into the periphery [10], act as antigen presenting cells (e.g. towards T cells) and hereby feed an auto-inflammatory circuit. Targeting of peripheral blood memory B cells might compromise such mechanisms, however, a combined modulation of both, intrathecal and peripheral B cell maturation seems to be a promising approach for treatment strategies in MS [10].

Certain limitations of our study have to be discussed. Although most of our findings on B cell distributions were in line with the literature, certain discrepancies can be found in comparison to our study and within previously published studies. These differences can, at least partially, be explained by the usage of different antibodies, gating strategies and definitions of B cell subtypes. In addition, differences in analyzed patient collectives in regard to disease course, disease activity and age might also account for diverse observations. The group of untreated MS patients mainly consisted of patients before receiving their first treatment cycle which resulted in a shorter disease duration for this group. Moreover, the lower number of patients with non-inflammatory neurological diseases was due to a lower number of patients matching the inclusion criteria. Sample acquisition was performed at two different sites which might have resulted in a slightly different sample analysis, however there was no statistical difference between the specific B cell subset from the two investigating sites. Lastly, this study was not interventional. Our analyses were performed during the routine diagnostic work-up for which reason the treatment duration ranged between 16–36 months and could possibly have affected the treatment effects on B cell subsets. However, consistent treatment effects are mostly detectable after 3–6 months and longitudinal data on treatment durations with more than 12 months, which could address possible habituation effects, are hardly available yet [26, 28, 33, 42, 43]. Future studies are necessary to follow treatment effects over a period longer than 12 months.

In conclusion we found evidence that several disease modifying treatments which exert immunomodulatory effects in the periphery target circulating memory B cells possibly contributing to the drugs’ mode of action in MS. For future studies it would be interesting to perform paralleled analyses of B cells in the peripheral blood and cerebrospinal fluid compartment in terms of B cell subtypes, clonality and functional aspects in order to gain further insights into pathogenic driving forces of multiple sclerosis and effective strategies of immunomodulation.

## Supporting information

S1 Table (XLSX)
